# Porphyrinic Molecular Devices: Towards Nanoscaled Processes

**DOI:** 10.3390/ijms11041878

**Published:** 2010-04-26

**Authors:** Melissa J. Latter, Steven J. Langford

**Affiliations:** 1 Centre for Strategic Nano-fabrication, The University of Western Australia, Crawley, West Australia 6009, Australia; E-Mail: Melissa.Latter@uwa.edu.au; 2 School of Chemistry, Monash University, Clayton, Victoria 3800, Australia

**Keywords:** porphyrins, molecular devices, logic operations, electron transfer, molecular rotors, self-assembly

## Abstract

The structural, coordinative, photochemical and electrochemical properties of the porphyrin macrocycle that make them the functional element of choice in ubiquitous biological systems, e.g., chlorophyll, cytochrome P450 and hemoglobin, also contribute to making porphyrins and metalloporphyrins desirable in a “bottom-up” approach to the construction of nanosized devices. This paper highlights some recent advances in the construction of supramolecular assemblies based on the porphyrin macrocycle that display optically readable functions as a result of photonic or chemical stimuli.

## 1. Introduction

**Figure 1.**
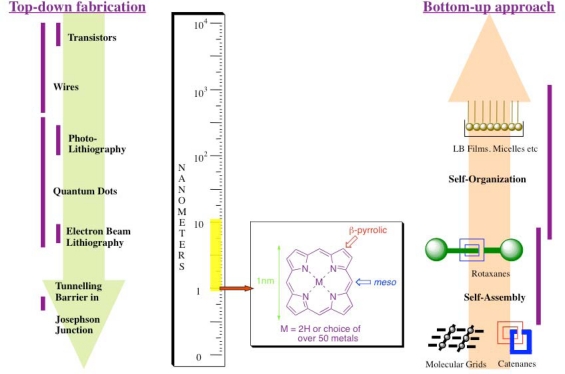
Comparison of two approaches for the preparation of devices at the nanometer level. The dimensions of the porphyrin macrocycle (nm) and its ability to be redox- and photo-active make it an ideal candidate for nanodevice fabrication.

The design and realization of a set of molecules capable of performing functions on the nanometer scale that mimic those executed by macroscopic electronic devices is of great scientific interest [1,2]. This area of science, broadly termed molecular electronics, utilizes a “bottom-up” approach by employing either single molecules or sets of complex molecular assemblies specifically designed for applications as switches, sensors, wires, shuttles, rectifiers, memory elements and logic gates on the nanometer level [3]. One of the driving forces behind this so-called “bottom-up” approach (Figure 1) arises from the complexities experienced by more traditional approaches of dismantling elemental arrays e.g., quantum tunneling effects, the localization of charge, and fabrication issues [3,4]. The “bottom-up” approach to assembling molecular devices is advantageous as it employs a discrete number of molecules designed to achieve a specific function, either on their own, or as components of a more complex supramolecular ensemble.

Supramolecular chemistry offers a paradigm shift for fundamental chemical research through a hybridization of organic, inorganic, theoretical and physical chemistry that focuses on the development of emerging technologies in all sciences and materials engineering. In essence, it is a bio-inspired discipline aimed at utilizing Natures concepts in the laboratory. While chemists have discovered many ways of bonding, the complexity and level of function of wholly-synthetic chemical systems is low compared to biological systems. For example, a comparison between palytoxin (a challenging molecule that has been prepared by total synthesis) and ribosomes as a biological example, reveal an awe-inspiring difference in the level of complexity. Interestingly, the higher order complexity of functioning biological systems requires a very basic (though catalyzed) set of chemical techniques. What is left is a void in which the interplay of chemistry and biology can be explored to form novel systems with a defined function that may mimic, or be different from natural systems. So often, the challenge lies not only in the preparation of suitable compounds but doing so in an efficient, economical and simplified fashion with the correct instruction for a given function, to generate an ordered construction or to control molecular motion [5,6].

Progress in the field of molecular devices will have important implications for molecular-based logic, energy storage and transduction, biomimetic engineering, therapeutics and a variety of sensory techniques including diagnostic tools. The active component(s) of a molecular device must display a number of important features *i.e.*, long-term stability, ease of fabrication and an addressable (fast) response. Electrochemical and photochemical inputs and outputs are among the easiest to interface to macroscopic systems, making them amenable to the multi-scale engineering required for the eventual creation of a pragmatic device. This view is strongly supported by theoretical calculations that suggest reversible electron transfer can serve as the basis for a molecular switch [7].

In terms of general device design, the following attributes for a light driven device are important:

- ET processes can effectively compete with deactivation of an excited state,
- the optical, guest binding and redox properties of the components will allow for the quantitative prediction of signaling parameters through complexation,
- the use of photoactive groups with high extinction coefficients and relatively low excitation energy means that readily accessible light sources can be used,
- there is a high sensitivity in detection, e.g., through fluorescence monitoring, which gives a quantitative “ON/OFF” switching ability per molecule (amenable to single molecule spectroscopy),
- the addition of receptor sites allow greater control of the electronic properties and provides an area for catalysis, and
- femtosecond resolution is possible through ET rates.

Furthermore, the ability of photosystems to allow electrons to move in one direction only means that in electronic terms, the system acts as a semiconducting diode. Connecting such diodes in combination may lead to the basis of novel computer technologies.

However, there are still a number of important and practical issues that need to be addressed before a pragmatic molecular device can be realized. One issue is based around the use of UV light as either an excitatory (input) or diagnostic (output) source. Such electromagnetic radiation is relatively high in energy, which will limit the lifetime of the device. Nature employs a strong visible light absorber as both electroactive and photoactive components within its functioning biological machinery. These absorbers, *i.e.*, porphyrin derivatives, have extinction coefficients (ɛ) of *ca*. 10^6^ dm^−1^M^−1^ in the region 400–440 nm, are fluorescent and their rich redox properties can be modulated effectively by inner peripheral coordination of over 50 different metal ions. Moreover, the favorable dimensions of the porphyrin core (1x1 nm), convenient synthesis and ease of functionalization at either the *meso*- or β-pyrrolic positions (Figure 1) are desirable properties for the construction of nanoscaled molecular devices. This paper concentrates on a limited but broad set of functions incorporating the porphyrin macrocycle that pertain to molecular device development from our research group.

## 2. Molecular Logic Devices — Chemical Sensing

**Figure 2.**
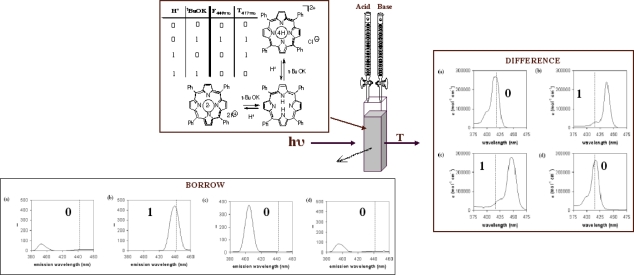
Demonstration of a molecular logic gate operation using the simple macrocyle tetraphenylporphyrin (TPP). Addition of acid, base, or both to a solution of TPP produces spectral changes, which can be detected by either transmission (difference output) or emission (borrow output). Interpretation of their conjoined use in Boolean algebra terms yields an example of a molecular half-subtractor.[9]

Applying the concept of binary processing to the molecular level requires a system that can be reversibly interconverted between two or more states to produce outputs of 0 (OFF) or 1 (ON). Simple logic operations such as YES, NOT, OR and AND can be easily demonstrated at the molecular level [6]. Complex operations such as *addition* and *subtraction,* however, require more complex operators - in particular an XOR logic gate [8]. By utilizing the amphiphilic nature of the inner periphery of 5,10,15,20-tetraphenylporphyrin (TPP) as well as changes in transmittance and emission at a particular wavelength we have been able to demonstrate (Figure 2) the chemical equivalents of XOR and INHIBIT logic gates [9]. The ability to multiconfigure the system, *i.e.*, detect transmittance and emission simultaneously led to this system being the first reported example of a molecular half-subtractor.

## 3. Nano-Scaled, Multicomponent Construction

**Figure 3.**
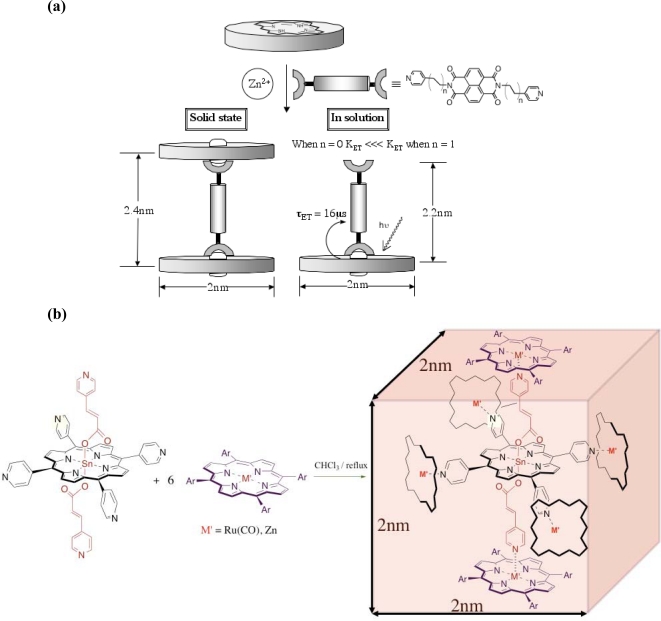
Assembly of multichromophoric constructs of nanoscale dimensions **(a)** the stoichiometry of the dyad assembly is phase dependant while the efficiency of the transduction, leading in one instant to a useful molecular battery, is component driven **(b)** the efficient self-assembly of seven components yields a molecular cube. Each face of the cube is constructed from a porphyrin macrocycle.

The construction of discrete molecular systems through the self-assembly of two or more components requires a high degree of molecular instruction [10] to be programmed into each component. This instruction dictates the connectivity, orientation and stability of the molecular assembly. Unlike a generic building block approach, molecular instruction places these (different) components in a precise, rather than random order, with respect to each other allowing for more sophisticated devices. This strategy becomes important for molecular assemblies that are designed for a specific supramolecular function, especially in applications related to energy transduction where the interaction between donor and acceptor moieties is critical. An example of our approach is shown in Figure 3. Here, we have employed the pentacoordinative property of Zn^2+^ and its propensity for pyridines to construct an efficient molecular battery, whose charge separated state upon photoinduced electron transfer lasts for microseconds (Figure 3a) [11]. Changes in the connectivity between the metalloporphyrin donor and the naphthalene diimide acceptor are able to expedite both electron transfer and charge recombination leading to an ability to tune the properties of the system. The ability to achieve lifetimes of the charge-separated state of microseconds is important for the ability to use this energy transduction process for useful chemical work (e.g., reduction).

The concept of a macroscopic building block employed to perform a structural function such as to build a wall, lay tiles *etc.,* can be applied to the nanometer level. In order to do so, molecular entities isostructural to bricks and blocks need to be efficiently synthesized. One approach (Figure 3b) is to essentially use a molecular block to lay down a pseudo-2D arrangement of light harvesting elements of solar energy conversion. The formation of the block can be achieved efficiently (>85% isolated yield) by a “one pot” self-assembly of *seven* components (shown in Figure 3b), and even *nine* components if the template is reduced to its components [12]. The template is itself a self-assembled entity where two pyridyl ligands are axially coordinated to the core *tetra*-pyridyl tin(IV) porphyrin providing the six coordination sites for coordination with either the zinc(II) or ruthenium(III) porphyrinic faces to yield a cubic geometry. Synthetic modification is possible either via covalent functionalization at the *meso-* or β-pyrrolic porphyrin positions or exploiting metal ion coordination to produce multi-chromophoric arrays. We are at present undertaking a covalent modification, post self-assembly to make the cube robust and to remove the template for host-guest applications. Towards this end we have also been successful in applying metathesis conditions to construct a covalently linked porphyrin containing square from a similar template-directed assembly strategy [13]. While this system serves primarily as proof-of-concept, this achievement is useful for furthering our knowledge in constructing nano-sized assemblies and we feel a significant bridging system for covalent modification opportunities of the more elaborate cubic geometry.

## 4. Molecular Rotors

Mechanical devices that function as a result of the rotary motion of some component are common within our macroscopic world (windmills, rotary motors, gyroscopes, propellers *etc.*) and biological apparatus (e.g., flagellum, ATP-ase) [14]. A molecular rotor can be defined as a molecular system in which a molecule or part of a molecule rotates against another part of the molecule [15] – in essence, requiring an axle or shaft and a component capable of rotation about the axis as a bear minimum. The nature of rotary motion can be classified in two ways, those in which the machine generates the rotary motion, and those in which the motion is generated by a stimuli including thermal energy. The macrocyclic structure of porphyrins offers an ideal platform with readout properties (fluorescence) while axial ligand(s) of metalloporphyrins provide axle components, facilitating the assembly of molecular rotors. Molecular rotors that are finding greatest utility are the so-called fluorescent molecular rotors (FMRs). The ability to match the internal rotation of a component against the excited state lifetime of another can be used for measuring, for example, in fluid dynamics where mechanical viscometry is not practical.

**Figure 4.**
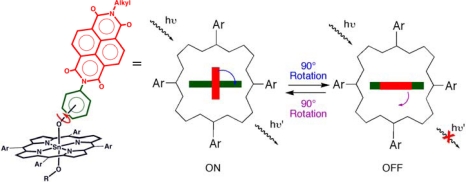
Schematic representation of tin(IV) porphyrin-phenolate complex that demonstrates rotor function. The rotation of the planar naphthalene diimde portion of the ligand (represented by red block) relative to the coordinated phenolate (green block) influences the fluorescent output of the tin(IV) porphyrin.

In the first example, that of an FMR, the porphyrin complex in Figure 4 acts as a fluorescent molecular rotor in which the conformational dynamics of the attached ligand controls the tin(IV) porphyrin fluorescence [16]. The fluorescent output also offers a means to probe performance, a feature of more importance when the rotor is operating as a component of a more complex functioning device. In essence, the rotational speed of the molecular rotor is proportional to the viscosity of the solute such as 1-octanol. As such, the viscosity of a solute can be measured through reading out the change in porphyrin fluorescence, which is a time-averaged property of two conformations. The schematic shown in Figure 4 depicts the point of rotation and the effect of the phenolate and naphthalene diimide in both coplanar and orthogonal arrangements. As a result of a redox event in the co-planar orientation, a strong fluorescence quenching of the tin(IV) porphyrin fluorescence is seen on account of an electron transfer process from the ligand to the tin(IV) porphyrin that is not seen in the orthogonal state.

**Figure 5.**
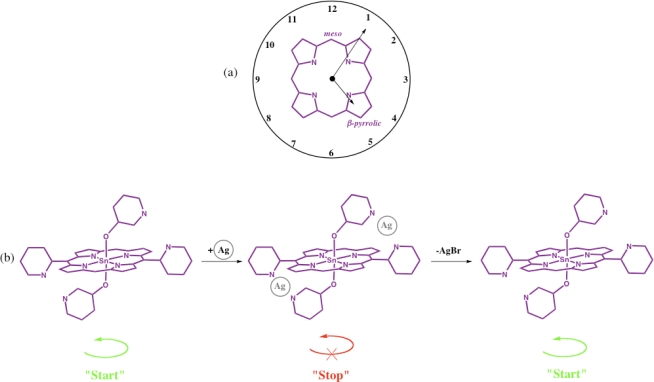
**(a)** The macrocyclic structure of the porphyrin can be envisaged as a clock face. The four *meso*- (corresponding to clock numbers 3, 6, 9 and 12) and eight β–pyrrolic positions (remaining clock numbers) may be readily functionalized. **(b)** Schematic demonstration of molecular control using metalloporphyrins to function as a stopwatch. Addition of silver ions provides the stimulus to block the free rotation of the axial ligands effectively “stopping” the stopwatch, which, upon addition of a further bromide salt, can reverse the complexation to “start” the stopwatch again.

In the second rotor example, the concept of a molecular ‘stopwatch’ has been presented utilizing the twelve peripheral positions of the porphyrin macrocycle (Figure 5a) to act as the twelve positions on a typical clock face [17]. Axial ligation allows the introduction of two pyridyl ligands that form the basis of the ‘hands’ of the clock. In this second generation timepiece a nanosized ‘stopwatch’ was constructed which can be stimulated to “start” and “stop” through additional complexation events (Figure 5b). In the initial state the axial ligands are free to rotate (“start” function) on the NMR timescale, but upon addition of silver ions, rotation is highly restricted leading to a “stop” function. The system can be activated again by the addition of a bromide salt to preferentially bind with the silver ions to allow free axial ligand rotation again (“start” function).

## 5. Self-Assembly on Surfaces

**Figure 6.**
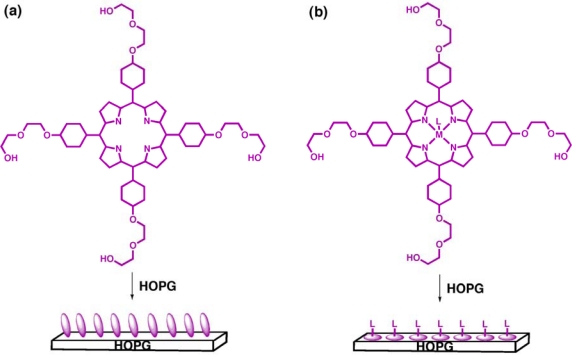
Schematic structure of “intelligent” porphyrins and their patterning on a graphite surface. **(a)** STM representation of free base porphyrins indicates face-on-face tilt packing along the surface. **(b)** Metallation introduces an axial ligand approximately orthogonal to the porphyrin macrocycle that patterns the surface in a planar, 2D layering. (L = pyridine).

Studying the assembly and deposition of chemical components on solid supports or surfaces also contributes to furthering our overall understanding towards controlling nanoscaled processes. One such approach utilizes “intelligent” porphyrins, where functionality is inbuilt, controlling the formation of arrays through molecular recognition events. The unusual self-association via hydrogen bonding of 2-ethoxyethanol substituents encouraged us to investigate the assembly modes of a porphyrin incorporating such pendant arms in the four *meso*-positions (Figure 6) on highly oriented pyrolytic graphite (HOPG) [18].

Using surface probe microscopy (SPM), ordered porphyrin layering on the surface was observed which was not molecularly resolved at ambient conditions but a defined patterning effect evident none the less. The favorable interactions between porphyrins produced face-on-face packing as shown schematically in Figure 6a. A small structural change, namely the introduction of an axial coordination site via metallation of the porphyrin macrocycle, has a pronounced affect on the observed patterning. The efficiency of face-on packing of the free base case is disrupted by the presence of the axial ligand and adsorption in a planar arrangement instead leads to two-dimensional layering on HOPG (Figure 6b). As shown above, it is proposed the axial ligand is not bridging the porphyrin and HOPG, rather directed on the exterior that provides a site for further manipulations to the surface properties and structure. This feature is particularly significant in molecular device fabrication so that various functions (e.g., switches, rotors, logic gates) can be built up from the surface assembly.

## 6. Conclusions

In summary, this paper illustrates some recent examples of manipulating the chemical and photochemical properties of porphyrins towards functioning molecules on the nanometer scale. For the most part, a self-assembly strategy inspired by Nature has been employed and the versatility of possible geometries and optical outputs using this macrocycle as a building block in developing nano-scaled devices has been demonstrated.
